# Comparing the Relative Importance of Predictors of Intention to Use Bicycles

**DOI:** 10.3389/fpsyg.2022.840132

**Published:** 2022-02-17

**Authors:** Valentina Baeli, Zira Hichy, Federica Sciacca, Concetta De Pasquale

**Affiliations:** Department of Educational Science, University of Catania, Catania, Italy

**Keywords:** intention to use a bicycle, Theory of Planned Behavior, habits, financial incentives, dominance analysis

## Abstract

The use of bicycles for active commuting is an important target to reach because of the importance of increasing physical activity among the population and improving the air quality in cities. Among the models that have been utilized in previous studies, the Theory of Planned Behavior (TPB) has shown good results in terms of the total variance obtained. However, establishing the relative importance of the TPB variables is difficult. In the present study, which was carried out in the Italian context, the authors sought to establish the weight of the proposed variables based on the dominance analysis approach. Considering the initiatives, which the Italian government carries out, and the particular period in which the study was developed, the authors included two variables in addition to the classical factors: financial incentives and daily commuting habits. A survey was administered to 294 Italians (222 females and 72 males, from 18 to 77 years old) through social networks from July to September 2020. The results have shown how the main predictor of bicycle use was use habits, followed almost at the same level by financial incentives and attitude, while norms and perceived behavioral control (PBC) present low relative importance among the variables considered. Limits of the study have been discussed, and suggestions for future research have been proposed.

## Introduction

In recent years, many institutions and governmental agencies have tried to improve the diffusion of alternative means of transportation, and an important target seems to be the diffusion of bicycle use for active commuting (ECF, 2018; Ministry of Environment, 2020). Understanding the factors that influence and determine the use of bicycles in cities could help to enhance air quality, reduce the number of automobiles in circulation, and improve the levels of physical activity in the population. Active travel is defined as methods of traveling to and from work that involve physical activity (walking and cycling; Jones and Ogilvie, 2012). According to this definition, active commuting can involve traveling only by walking or cycling, or traveling by walking or cycling in combination with motorized travel (e.g., using a combination of walking and car or train and cycling; Jones and Ogilvie, 2012). Psychology has provided a great number of useful models and tools to understand the factors that determine this behavior, especially considering the high complexity of the factors involved [for a review of the most widely used models, see Lanzini and Khan (2017)]. One model, in particular, has shown excellent fit in identifying key variables for active commuting behaviors: the Theory of Planned Behavior (TPB, Ajzen, 1991). After the dispersal of COVID-19, the number of studies on bicycle use for active commuting in the Italian context has increased (e.g., Torrisi et al., 2021); however, none of these studies have focused on the TPB framework. In a recent review, Caballero et al. (2019) observed that the model explained 25–73% of the variance in intention to use a bicycle. The model assumes that behavioral intention is determined by three fundamental factors: attitudes, subjective norms, and perceived behavioral control (PBC). Moreover, studies have indicated that the role of habits in determining the intention to use a bicycle is unclear (de Bruijn et al., 2009; Heinen et al., 2011; Rondinella et al., 2012; Muñoz Lopez et al., 2013; Caballero et al., 2014), as well as the role of financial incentives in decision-making processes regarding commute mode choice (Bamberg and Schmidt, 2000; Riggs, 2017). The present study, which was carried out in the Italian context, aims to investigate the relative importance of TPB variables, habits, and financial incentives in determining the intention to use a bicycle for active commuting.

### Theory of Planned Behavior and Commute Mode Choice

Since its introduction, the TPB (Ajzen, 1991) has been used to understand behavioral intentions in various areas, such as consumer choices, environmentally supportive behaviors, and health behaviors (Harland et al., 1999; Sheeran et al., 2001; Garcìa et al., 2019; Grilli and Notaro, 2019; Coşkun and Özbük, 2020; Zhang and Li, 2020); moreover, the TPB is one of the most widely used psychological models to study commuting behavior [for a review, see Jakovcevic et al. (2015)]. The theory states that subjects rationally make decisions based on a cost-benefit calculation. According to the TPB, behaviors depend on three variables: *attitude*, which is positive or negative toward the behavior; *subjective norms*, which represent perceived social pressure; and *perceived behavioral control*, which represents the degree of control over behavior. Together, these variables determine *intention*, the closest predictor of behavior (Ajzen, 2005). Positive attitudes, good subjective norms, and good control levels determine intention (Ajzen, 2005). The model has been widely applied to explain the use of the bicycle as a means of transport in different contexts and age groups (Bamberg, 2006; Haustein and Hunecke, 2007; de Bruijn et al., 2009; Milkovic and Stambuk, 2015; Frater et al., 2017; Acheampong and Siiba, 2018; Caballero et al., 2019) sometimes even in expanded versions to increase its predictive power (Lois et al., 2015). Recently, Caballero et al. (2019) argued that estimating the relative importance of model variables was difficult because of the different research methodologies used. According to the above discussion, this study uses TPB as the theoretical framework to analyze the use of the bicycle as a means of transport for daily commuting. Indeed, as we will see, attitudes, subjective norms, and PBC are all variables that influence intentions to use the bicycle.

#### Attitudes

According to Eagly and Chaiken (1993, p. 1), attitude is “a psychological tendency that is expressed by evaluating a particular entity with some degree of favor or disfavor.” Many research studies on commute mode choice have observed that attitudes toward bicycles were an important variable in determining the intention to use a bicycle or bike-sharing system or to cycle to school (Dill and Voros, 2007; Heinen et al., 2011; Milkovic and Stambuk, 2015; Frater et al., 2017; Yu et al., 2018). The results show that positive attitudes determine the intention to use a bicycle over long distances compared to short distances (Heinen et al., 2011), while a relationship between positive attitudes and greater PBC has been observed (de Souza et al., 2014). Therefore, as it appears from previous studies, attitude is one of the most influential variables in determining intentions to use a bike.

#### Subjective Norms

Considering Ajzen’s (1991) definition, the subjective norm is the perception of significant others’ judgment about a behavior. Some studies indicated that subjective norms were the main predictor of the intention to cycle to school in groups of young students (Frater et al., 2017; Fitch et al., 2019). Some authors have observed that subjective norms had a greater effect than attitudes on the intention to use a bicycle to travel short distances (Heinen et al., 2011). From a theoretical and practical perspective, subjective norms have been conceptualized as prescriptive (injunctive) or descriptive norms (Cialdini et al., 1990). The distinction describes different shades in the normative influence: prescriptive norms define what is commonly accepted and valued by a social group, whereas descriptive norms refer to the extent to which others are engaging in a given behavior. Obviously, the two types may frequently overlap. As you can see, even in the case of subjective norms, a strong tie between this variable and intentions of using the bike appears.

#### Perceived Behavioral Control

PBC is the personal perception of the ease or difficulty in carrying out a particular behavior (Ajzen, 2005). In commute mode choice studies, the difference between internal and external control proposed by Manstead and van Eekelen (1998) is useful. Internal control reflects the ability of the individual to act, while external control concerns the perception of available resources and the objective difficulties of the task (Terry et al., 1993; Manstead and van Eekelen, 1998). Many studies have observed how PBC^1^ is a fundamental variable in determining the intention to use a bicycle as a means of transport (Caballero et al., 2014; de Souza et al., 2014; Acheampong and Siiba, 2018; Si et al., 2020). Moreover, gender differences were highlighted: women with higher PBC showed a greater intention to use bike-sharing (Cai et al., 2019) and perceived existing barriers more strongly than men (Titze et al., 2007). However, the role of PBC in relation to the objective presence of infrastructure is unclear. Indeed, some studies have observed that although the objective presence of infrastructure has increased the likelihood of cycling, it is not a determining factor, with cycling considered an individual choice independent of the infrastructure conditions (Moudon et al., 2005; Parkin et al., 2008); however, when infrastructure is objectively present, internal factors seem decisive (De Geus et al., 2008). Hence, it is evident that the PBC plays an important role in determining the intentions of using the bike, even if it can be influenced by contextual variables (e.g., presence of infrastructure).

### Habits

Several definitions of habit are available in the literature (Wood and Neal, 2007; Gardner, 2015): habit is generally an expression of learned, frequently implemented, and automated behavior. The habit of using a means of transport is an important predictor at the behavioral level, even if the literature has highlighted a decrease in the strength of intention when behavior becomes habitual (de Bruijn et al., 2009; Heinen et al., 2011; Caballero et al., 2014). However, the high frequency of bicycle use correlates with a positive perception of the behavior (Rondinella et al., 2012). Another study, carried out in the Spanish context, showed that cycling habits were the strongest predictor of bicycle use, while other psychological variables, such as attitude, played a less important role (Muñoz Lopez et al., 2013). From these studies, it is clear that habits can be ambivalent in determining the intentions of using the bicycle as a means of transport for daily commuting; therefore, it is important to understand their role.

### Financial Incentives

The use of incentives to promote healthy behavior is not a novelty (see for a review, Mantzari et al., 2015). In the context of sustainable mobility, there is a broad literature on the use of incentives; indeed, various studies have explored their use in changing commute mode (Barf et al., 1982; Bamberg, 2006; Thøgersen, 2009; Martin et al., 2012; Winters et al., 2017; de Krujif et al., 2018; Máca et al., 2020; Ciccone et al., 2021). The kind of incentives explored in the studies is various, such as monetary incentives to buy a bicycle, free use of e-bikes, vouchers, different forms of gifts, and monetary rewards through smartphone apps (see for a review, Mantzari et al., 2015). Even if there are many studies on the topic, results about the effects of incentives in changing commute mode are not clear, as well as the relationship between incentives and other variables. For example, incentives seem to have an impact on the decision to travel by bike or other sustainable modes of travel (de Krujif et al., 2018; Moser et al., 2018; Máca et al., 2020; Ciccone et al., 2021); however, it is not clear enough if the effect is long-lasting. Furthermore, is not clear the relationship between financial incentives and psychological factors. For example, Hunecke et al. (2001) observed similar but independent effects of free tickets and personal norms on switching to a more sustainable commute mode, whereas Riggs (2017) observed that moral values have stronger effects than financial factors on behavioral change. Moreover, some authors assessed that incentives could have no or few effects when realistic alternatives are unavailable (Bamberg and Schmidt, 2000) or when bike use is particularly valued (Riggs, 2017); while other authors assessed that incentives could have more power for particular subjects (Huang et al., 2018). Furthermore, considering the interplay between attitude and incentives within the TPB framework, Bamberg and Schmidt (1998) observed that incentives affect behavior through attitude. At the same time, previous literature has explored the relationship between different kinds of incentives and habits: these studies showed that incentives could be a means to create new habits and break older ones, or to create new habits when people are more reactive to intervention (Bamberg and Schmidt, 2003; Bamberg, 2006; Thøgersen, 2009; Moser et al., 2018). Finally, Savelkoul and Peutz (2017) observed that financial incentives added a non-significant percentage of variance in bicycle commuting intention. As you can see, the results relating to the effects of incentives are discordant; for this reason, it is important to include this variable in the study of intentions to use the bike.

### Dominance Analysis

Dominance analysis (Budescu, 1993; Azen and Budescu, 2003) is an extension of multiple regression aimed to determine the importance of one predictor over the others by examining the *R*^2^ across all possible subsets of independent variables. This analysis can be used for confirming or exploring which predictor is more important. Azen and Budescu (2003) proposed three types of dominance: complete, conditional, and general. In the complete dominance, a predictor’s additional contribution (the increase in *R*^2^ resulting from adding it to the regression model) to each subset model is greater than that of the other variable. Nevertheless, because complete dominance is difficult to reach, to reduce the occurrence of undetermined dominance, Azen and Budescu (2003) proposed two supplementary but weaker levels of dominance. In conditional dominance, a predictor’s average of additional contributions within models of the same size (that is, the same number of predictors) is greater than that of the other predictor. In the general dominance, a predictor’s average of all additional contributions is greater than that of the other predictor. Furthermore, dominance analysis resolves the problem of multicollinearity. Indeed, it examines the change in *R*^2^ that results from adding an independent variable to all possible subset regression models providing the context for comparison (Barni, 2015). Furthermore, multiple regression considers the contribution of a predictor in terms of increasing the explained variance of the dependent variable in the presence of other predictors. This procedure is correct from a statistical point of view, but it can be far from reality in some research fields. For example, Johnson and LeBreton (2004), in the field of customer satisfaction, argue that multiple regression inadequately captures reality because customers do not consider the incremental satisfaction that derives from one variable while maintaining the other constants. Conversely, when customers assess their level of satisfaction, they assign a weight to each predictor (Tonidandel and LeBreton, 2011). The same can be said in the case of intentions to use the bike: indeed, it is more likely that in deciding to use the bike for daily commuting, people consider all the factors involved (e.g., presence of infrastructures, the opinion of friends and relatives) and attribute a weight to each one.

Dominance analysis was employed as a valuable statistical tool to determine the importance of predictors in various studies conducted in various psychological fields, such as cognition (Gellersen et al., 2021), customer satisfaction (Garver and Williams, 2019), family (Oyarzún-Farías et al., 2021), personality (Duan et al., 2021), organization (Simonet et al., 2019), and religion (Hichy et al., 2014). All those studies have identified the variables that influence the criterion; moreover, they have the advantage of establishing which variable is the most important and, therefore, on which variable it is convenient to intervene.

Regarding environment psychology, there are no specific studies using dominance analysis; however, a study on automated public transportation (Bernhard et al., 2020) analyzed determinants of intention to use autonomous public transport applying both multiple regression and dominance analysis. Results showed that performance expectancy, effort expectancy, age, and experience were significant predictors of intention to use an autonomous minibus. However, age and experience have small dominance weights (meaning they accounted for a small amount of variance), while the most important predictor was performance expectancy, followed by effort expectancy. These results suggest that in order to increase the intention of using automated public transportation should improve the performance expectancy of users.

### Present Study

This study aims to investigate the relative importance of attitude, subjective norms (descriptive and prescriptive), PBC, habits, and financial incentives as predictors of intention to use a bicycle for daily commuting. As we have seen, there is extensive literature on all the variables taken into consideration; however, the results are heterogeneous, and it is not clear the importance of the single variables in determining the intentions of using the bike (Caballero et al., 2019). For example, the interplay between external incentives and other psychosocial variables, such as social norms or values, is unclear, as well as the dominance of one factor over the others (Hunecke et al., 2001; Riggs, 2017).

Most of the researchers that analyzed variables influencing the use of bicycles for daily commuting have used the multiple regression approach, which aims to explain the dependent variable from several independent variables, regardless of their predictive power. In this study, we applied the dominance analysis approach (Budescu, 1993; Azen and Budescu, 2003) that allows us to determine the dominance of one predictor over the others by assessing its contribution to the prediction of the dependent variable. As we have seen, using dominance analysis allows establishing which predictor affects the dependent variable and understanding which predictor is the most important. This aspect, of course, plays an important role when it needs to define the most effective variables to encourage people to use the bike. Furthermore, this procedure is particularly useful in studies, such as those related to TPB, having multiple independent variables for which the problem of multicollinearity can arise.

#### Context of the Study

The transition to sustainable mobility is one of the fundamental targets of the Italian government and the European Union (ECF, 2020; Ministry of Environment, 2020; Ministry of Transportation, 2020). With the spread of the COVID-19 emergency, the Italian government allocated funds to buy a bicycle, giving people the possibility of accessing the “mobility bonus.” The beneficiaries eligible for the bonus were adults residing in municipalities with a population greater than 50,000 inhabitants, in regional capitals and provincial capitals (even with less than 50,000 inhabitants), in metropolitan cities, and municipalities belonging to metropolitan cities (even under 50,000 inhabitants). The mobility bonus was a contribution equal to 60% (not exceeding 500 euros) of the cost incurred for purchasing a bicycle or other vehicle for personal mobility with electric propulsion (e.g., hoverboards). The bonus was valid for purchases made between May 4th and December 31st, 2020.

## Materials and Methods

### Participants and Procedures

Participants were an opportunity sample of 294 Italians (222 females and 72 males) aged between 18 and 77 years (*M* = 39.74, *SD* = 13.41) who were asked to complete an online questionnaire (see Supplementary Material) posted on principal social networks from July to September 2020. Regarding residence, 165 participants resided in Northern, 62 in Central, and 67 in Southern Italy and Islands (see Supplementary Material, for detailed provinces of residence). To establish the sample size, we used Soper’s (2021) calculator for multiple regression. With six independent variables, the minimal sample size required to reach a power of 0.80, a probability level of 0.05 and effect size of 0.15 (small *f* = 0.02, medium *f* = 0.15, and large *f* = 0.35), was 97 participants. All participants were informed that their responses would remain confidential.

### Measures

#### Descriptive Norms

To measure descriptive norms, one item was used (“How many of your friends/relatives use a bike to travel around the city?” see Passafaro et al., 2014). Participants expressed their opinion on a 7-point scale (1 = none, 2 = few, 3 = some, 4 = half, 5 = many, 6 = almost all, 7 = all).

#### Prescriptive Norms

To measure prescriptive norms, one item was used (“People important to me think I should ride a bike more often”; Passafaro et al., 2014). Participants expressed their opinion on a 7-point scale ranging from 1 (strongly disagree) to 7 (strongly agree).

#### Attitude Toward Behavior

To measure attitude toward using a bike for daily commuting, nine 7-point bipolar scales were used (e.g., “difficult/easy,” Passafaro et al., 2014). The alpha value was 0.83.

#### Perceived Behavioral Control

To detect behavioral control, two items conceived by the authors were used (e.g., “I see many difficulties in riding a bike”). For each item, participants expressed their opinion on a 7-point scale ranging from 1 (strongly disagree) to 7 (strongly agree). The alpha value was 0.62.

#### Habits

To assess the habits related to bike use before the lockdown, participants were asked about their frequency of bike use (“Before the lockdown, how often did you use your bicycle for daily commuting”). Participants answered on a 7-point scale (1 = never, 2 = rarely, 3 = sometimes, 4 = half the time, 5 = often, 6 = almost always, 7 = always). The authors created the proposed question considering the pandemic context in which many habits have been stopped for some time.

#### Financial Incentives

To measure the effect of financial incentives, two items, conceived by the authors considering the initiatives proposed by the Italian government, were used (e.g., “Government incentives for purchasing a bike encourage me to buy it”). For each item, participants expressed their opinion on a 7-point scale ranging from 1 (strongly disagree) to 7 (strongly agree). The alpha value was 0.83.

#### Intention

To evaluate the intention to use a bike, one item created by the authors was used (“I intend to start using a bike for daily commuting”). Participants answered on a 7-point scale ranging from 1 (strongly disagree) to 7 (strongly agree).

## Results

Table 1 shows the means, standard deviations, and correlations between the investigated variables. The results showed that for descriptive norms, participants did not have many models that used a bike for daily commuting, while for prescriptive norms, a moderate level was observed. The attitude toward the behavior is slightly positive, whereas the perceived behavior control is low. With regard to habits, participants rarely used bikes before lockdown. Concerning financial incentives, the analysis revealed a low propensity for their use and low intentions to begin to use a bike for daily commuting. Regarding correlations, intention was positively correlated with all predictors considered; moreover, all predictors were positively correlated with each other except for habits and prescriptive norms, financial incentives and both descriptive norms and behavioral control.

**TABLE 1:** Means, standard deviations, regression coefficients, and correlations.

		*M*	*SD*	1	2	3	4	5	6	7
1	Descriptive norms	2.04	0.99	1						
2	Prescriptive norms	3.55	1.69	0.141*	1					
3	Attitude	4.37	1.07	0.353**	0.269**	1				
4	Behavioral control	3.03	1.34	0.323**	0.227**	0.515**	1			
5	Habits	1.97	1.27	0.363**	0.106	0.582**	0.351**	1		
6	Financial incentives	3.27	1.76	0.110	0.194**	0.223**	0.020	0.154**	1	
7	Intentions	3.51	1.90	0.221**	0.327**	0.537**	0.308**	0.548**	0.419**	1

*p < 0.05. **p < 0.01.

To determine the relative importance of the variables considered in this study (descriptive norms, prescriptive norms, attitude, behavioral control, habits, and financial incentives) in predicting intention to use a bike, a dominance analysis was performed (Azen and Budescu, 2003).

Table 2 shows the 2^6^ = 64 subset models evaluated and their corresponding *R*^2^, additional contributions of excluded variables (columns labeled *X*_*i*_), and the average of additional contributions to all subset models of a given size (rows labeled *k*).

**TABLE 2:** Additional contributions of predictors across all subset regression models.

		Additional contribution of:						
Model		*R* ^2^	*X*_1_	*X*_2_	*X*_3_	*X*_4_	*X*_5_	*X*_6_
*k* = 0 average		0	0.0489	0.1070	0.2877	0.0950	0.3007	0.1759
*X*_1_	Descriptive norms	0.0489		0.0893	0.2399	0.0626	0.2523	0.1580
*X*_2_	Prescriptive norms	0.1070	0.0312		0.2168	0.0577	0.2669	0.1317
*X*_3_	Attitude	0.2877	0.0012	0.0361		0.0014	0.0843	0.0947
*X*_4_	Behavioral control	0.0950	0.0165	0.0697	0.1941		0.2210	0.1707
*X*_5_	Habits	0.3007	0.0006	0.0733	0.0714	0.0153		0.1149
*X*_6_	Financial incentives	0.1759	0.0311	0.0628	0.2066	0.0898	0.2397	
*k* = 1 average			0.0161	0.0662	0.1858	0.0454	0.2128	0.1340
*X*_1_ *X*_2_		0.1382			0.1862	0.0385	0.2357	0.1219
*X*_1_ *X*_3_		0.2889		0.0355		0.0010	0.0839	0.0941
*X*_1_ *X*_4_		0.1115		0.0652	0.1783		0.2044	0.1612
*X*_1_ *X*_5_		0.3012		0.0727	0.0716	0.0148		0.1144
*X*_1_ *X*_6_		0.2070		0.0532	0.1760	0.0658	0.2086	
*X*_2_ *X*_3_		0.3238	0.0006			0.0003	0.0920	0.0804
*X*_2_ *X*_4_		0.1647	0.0120		0.1594		0.2141	0.1361
*X*_2_ *X*_5_		0.3739	0.0000		0.0419	0.0049		0.0870
*X*_2_ *X*_6_		0.2386	0.0215		0.1656	0.0622	0.2222	
*X*_3_ *X*_4_		0.2891	0.0008	0.0350			0.0832	0.0986
*X*_3_ *X*_5_		0.3721	0.0007	0.0438		0.0003		0.0894
*X*_3_ *X*_6_		0.3825	0.0006	0.0217		0.0052	0.0790	
*X*_4_ *X*_5_		0.3160	0.0000	0.0629	0.0564			0.1181
*X*_4_ *X*_6_		0.2657	0.0071	0.0351	0.1220		0.1684	
*X*_5_ *X*_6_		0.4156	0.0000	0.0453	0.0459	0.0185		
*k* = 2 average			0.0043	0.0470	0.1203	0.0211	0.1592	0.1101
*X*_1_ *X*_2_ *X*_3_		0.3244				0.0002	0.0931	0.0801
*X*_1_ *X*_2_ *X*_4_		0.1767			0.1479		0.2026	0.1296
*X*_1_ *X*_2_ *X*_5_		0.3740			0.0435	0.0053		0.0872
*X*_1_ *X*_2_ *X*_6_		0.2601			0.1444	0.0462	0.2011	
*X*_1_ *X*_3_ *X*_4_		0.2899		0.0347			0.0833	0.0979
*X*_1_ *X*_3_ *X*_5_		0.3728		0.0447		0.0004		0.0899
*X*_1_ *X*_3_ *X*_6_		0.3830		0.0215		0.0048	0.0796	
*X*_1_ *X*_4_ *X*_5_		0.3160		0.0633	0.0572			0.1189
*X*_1_ *X*_4_ *X*_6_		0.2728		0.0335	0.1150		0.1621	
*X*_1_ *X*_5_ *X*_6_		0.4156		0.0456	0.0471	0.0193		
*X*_2_ *X*_3_ *X*_4_		0.3241	0.0005				0.0918	0.0831
*X*_2_ *X*_3_ *X*_5_		0.4158	0.0017			0.0001		0.0737
*X*_2_ *X*_3_ *X*_6_		0.4042	0.0003			0.0030	0.0853	
*X*_2_ *X*_4_ *X*_5_		0.3788	0.0005		0.0371			0.0907
*X*_2_ *X*_4_ *X*_6_		0.3008	0.0055		0.1064		0.1687	
*X*_2_ *X*_5_ *X*_6_		0.4609	0.0003		0.0286	0.0086		
*X*_3_ *X*_4_ *X*_5_		0.3723	0.0009	0.0436				0.0917
*X*_3_ *X*_4_ *X*_6_		0.3877	0.0001	0.0195			0.0763	
*X*_3_ *X*_5_ *X*_6_		0.4614	0.0012	0.0281		0.0026		
*X*_4_ *X*_5_ *X*_6_		0.4341	0.0008	0.0354	0.0299			
*k* = 3 average			0.0012	0.0370	0.0757	0.0090	0.1244	0.0943
*X*_1_ *X*_2_ *X*_3_ *X*_4_		0.3246					0.0929	0.0827
*X*_1_ *X*_2_ *X*_3_ *X*_5_		0.4175				0.0000		0.0741
*X*_1_ *X*_2_ *X*_3_ *X*_6_		0.4045				0.0027	0.0871	
*X*_1_ *X*_2_ *X*_4_ *X*_5_		0.3793			0.0382			0.0917
*X*_1_ *X*_2_ *X*_4_ *X*_6_		0.3063			0.1009		0.1647	
*X*_1_ *X*_2_ *X*_5_ *X*_6_		0.4612			0.0304	0.0097		
*X*_1_ *X*_3_ *X*_4_ *X*_5_		0.3732		0.0443				0.0928
*X*_1_ *X*_3_ *X*_4_ *X*_6_		0.3878		0.0194			0.0782	
*X*_1_ *X*_3_ *X*_5_ *X*_6_		0.4627		0.0289		0.0033		
*X*_1_ *X*_4_ *X*_5_ *X*_6_		0.4348		0.0361	0.0311			
*X*_2_ *X*_3_ *X*_4_ *X*_5_		0.4159	0.0016					0.0745
*X*_2_ *X*_3_ *X*_4_ *X*_6_		0.4072	0.0001				0.0832	
*X*_2_ *X*_3_ *X*_5_ *X*_6_		0.4895	0.0021			0.0008		
*X*_2_ *X*_4_ *X*_5_ *X*_6_		0.4695	0.0015		0.0209			
*X*_3_ *X*_4_ *X*_5_ *X*_6_		0.4640	0.0020	0.0263				
*k* = 4 average			0.0014	0.0310	0.0443	0.0033	0.1012	0.0831
*X*_1_ *X*_2_ *X*_3_ *X*_4_ *X*_5_		0.4175						0.0755
*X*_1_ *X*_2_ *X*_3_ *X*_4_ *X*_6_		0.4072					0.0858	
*X*_1_ *X*_2_ *X*_3_ *X*_5_ *X*_6_		0.4916				0.0014		
*X*_1_ *X*_2_ *X*_4_ *X*_5_ *X*_6_		0.4709			0.0220			
*X*_1_ *X*_3_ *X*_4_ *X*_5_ *X*_6_		0.4660		0.0270				
*X*_2_ *X*_3_ *X*_4_ *X*_5_ *X*_6_		0.4903	0.0026					
*k* = 5 average			0.0026	0.0270	0.0220	0.0014	0.0858	0.0755
*X*_1_ *X*_2_ *X*_3_ *X*_4_ *X*_5_ *X*_6_		0.4930						
Overall average			0.0124	0.0525	0.1226	0.0292	0.1640	0.1121

*R^2^ = variance in Y explained by the model appearing in the corresponding row. Columns labeled X_1_—X_6_ represent the additional contributions to the explained variance gained by adding the variable to the model. Rows labeled k = (number) average represent the average additional contribution within each model size. Empty cells indicate that data are not applicable.*

To establish complete dominance, the additional contribution of one variable must be compared with the additional contribution of another variable across all subset models. For example, to establish complete dominance between attitude and habits, you have to compare the columns of Table 2, respectively labeled *X*_3_ and *X*_5_. If the additional contribution of one predictor is always greater than that of the other predictor, then it can be said that one predictor completely dominates the other. Results indicate that habits (*X*_5_) completely dominate descriptive (*X*_1_) and prescriptive norms (*X*_2_), attitude (*X*_3_), and behavioral control (*X*_4_); financial incentives (*X*_6_) completely dominate descriptive norms (*X*_1_), prescriptive norms (*X*_2_), and behavioral control (*X*_4_); attitude (*X*_3_) completely dominates descriptive norms (*X*_1_) and behavioral control (*X*_4_); prescriptive norms (*X*_2_) completely dominate descriptive norms (*X*_1_). In all other cases, complete dominance cannot be established; indeed, the relative importance of variables changes based on the subset model used for making the comparison.

To establish conditional dominance, the average additional contribution of one predictor within each model size must be compared with the average additional contribution of another variable. For example, to establish conditional dominance between attitude and habits, you must compare the entry under columns labeled *X*_3_ and *X*_5_ in each *k* row of Table 2. If the average contribution of one predictor is greater than that of the other predictor for each model size, then it can be said that one predictor conditionally dominates the other. Results indicate that prescriptive norms (*X*_2_) dominate behavioral control (*X*_4_) while habits (*X*_5_) dominate financial incentives (*X*_6_); for the remaining case, conditional dominance still cannot be established. Indeed, the average additional contribution, within each model size, in some cases is greater for one predictor while in other cases is greater for another.

To establish general dominance, one predictor’s overall averaged additional contribution must be compared with the overall averaged additional contribution of another predictor. For example, to establish general dominance between attitude and habits, you must compare the entry under columns labeled *X*_3_ and *X*_5_ in the last row of Table 2 (Overall average). If the overall averaged contribution of one predictor is greater than that of the other predictor, then it can be said that one predictor generally dominates the other. Results indicate that attitude (*X*_3_) dominates prescriptive norms (*X*_2_) and financial incentives (*X*_6_) while behavioral control (*X*_4_) dominates descriptive norms (*X*_1_).

Taken together, these results indicate that habits are the stronger predictor of intention to use a bicycle for daily commuting, followed by attitude and incentives, which have almost the same weight, while prescriptive norms, PBC, and descriptive norms are the weaker predictors.

Following Azen and Budescu (2003), to assess the generality and robustness of the obtained results, the degree to which the dominance pattern obtained in the sample is reproduced in 1,000 bootstrap samples was measured (Table 3). If the mean of dominance values (mean of *D*_*ij*_) is closer to one, *X*_*i*_ dominates *X*_*j*_; if the mean of dominance is closer to zero, *X*_*j*_ dominates *X*_*i*_; if the mean of dominance is closer to 0.5 is not possible to establish the dominance. Another method of evaluating the generality and robustness of the results is the reproducibility, which could be intended as the confidence level; for example, if the reproducibility is 0.96, the result was replicated in 96% of bootstrap samples, then we can be 96% confident in concluding that a predictor dominates another in the population.

**TABLE 3:** Dominance values in the sample (*n* = 294) and their means, standard errors, probabilities, and reproducibility over 1,000 bootstrap samples.

*X*_*i*_	*X*_*j*_	*D*_*ij*_	Mean of *D*_*ij*_	*SE* of *D*_*ij*_	*P*_*ij*_	*P*_*ji*_	*P*_*noij*_	Reproducibility
**Complete dominance**								
2	1	1.0	0.8805	0.213	0.761	0.000	0.239	0.761
2	4	0.5	0.5870	0.194	0.177	0.003	0.820	0.820
3	1	1.0	0.9685	0.122	0.937	0.000	0.063	0.937
3	2	0.5	0.6565	0.232	0.313	0.000	0.687	0.687
3	4	1.0	0.9595	0.136	0.919	0.000	0.081	0.919
4	1	0.5	0.5585	0.161	0.117	0.000	0.883	0.883
5	1	1.0	1.0000	0.000	1.000	0.000	0.000	1.000
5	2	1.0	0.9520	0.147	0.904	0.000	0.096	0.904
5	3	1.0	0.7830	0.277	0.597	0.031	0.372	0.597
5	4	1.0	0.9975	0.035	0.995	0.000	0.005	0.995
5	6	0.5	0.6305	0.256	0.296	0.035	0.669	0.669
6	1	1.0	0.9955	0.047	0.991	0.000	0.009	0.991
6	2	1.0	0.9180	0.198	0.846	0.010	0.144	0.846
6	3	0.5	0.5340	0.164	0.090	0.022	0.888	0.888
6	4	1.0	0.9580	0.139	0.916	0.000	0.084	0.916
**Conditional dominance**								
2	1	1.0	0.9185	0.185	0.837	0.000	0.163	0.837
2	4	1.0	0.7700	0.283	0.576	0.036	0.388	0.576
3	1	1.0	0.9705	0.118	0.941	0.000	0.059	0.941
3	2	0.5	0.6975	0.245	0.395	0.000	0.605	0.605
3	4	1.0	0.9665	0.125	0.933	0.000	0.067	0.933
4	1	0.5	0.7005	0.266	0.422	0.021	0.557	0.557
5	1	1.0	1.0000	0.000	1.000	0.000	0.000	1.000
5	2	1.0	0.9855	0.087	0.972	0.001	0.027	0.972
5	3	1.0	0.7885	0.281	0.613	0.036	0.351	0.613
5	4	1.0	0.9985	0.027	0.997	0.000	0.003	0.997
5	6	1.0	0.7270	0.323	0.538	0.084	0.378	0.538
6	1	1.0	0.9965	0.042	0.993	0.000	0.007	0.993
6	2	1.0	0.9255	0.222	0.886	0.035	0.079	0.886
6	3	0.5	0.5370	0.172	0.099	0.025	0.876	0.876
6	4	1.0	0.9625	0.132	0.925	0.000	0.075	0.925
**General dominance**								
2	1	1.0	0.9800	0.140	0.980	0.020	0.000	0.980
2	4	1.0	0.8250	0.380	0.825	0.175	0.000	0.825
3	1	1.0	1.0000	0.000	1.000	0.000	0.000	1.000
3	2	1.0	0.9780	0.147	0.978	0.022	0.000	0.978
3	4	1.0	0.9970	0.055	0.997	0.003	0.000	0.997
3	6	1.0	0.5100	0.500	0.510	0.490	0.000	0.510
4	1	1.0	0.9010	0.299	0.901	0.099	0.000	0.901
5	1	1.0	1.0000	0.000	1.000	0.000	0.000	1.000
5	2	1.0	0.9950	0.071	0.995	0.005	0.000	0.995
5	3	1.0	0.8630	0.344	0.863	0.137	0.000	0.863
5	4	1.0	1.0000	0.000	1.000	0.000	0.000	1.000
5	6	1.0	0.7570	0.429	0.757	0.243	0.000	0.757
6	1	1.0	1.0000	0.000	1.000	0.000	0.000	1.000
6	2	1.0	0.9510	0.216	0.951	0.049	0.000	0.951
6	4	1.0	0.9960	0.063	0.996	0.004	0.000	0.996

*X_i_ and X_j_, variables that are compared (1, descriptive norms; 2, prescriptive norms; 3, attitude; 4, behavioral control; 5, habits; and 6, financial incentives); D_ij_, dominance values in the sample (1, X_i_ dominates X_j_; 0, X_j_ dominates X_i_; 0.5, dominance cannot be established); Mean of D_ij_, average of D_ij_ value over the bootstrap samples; SE of the D_ij_, standard error (variability) of the D_ij_ values over the bootstrap samples; P_ij_ proportion of bootstrap samples in which X_i_ dominated X_j_, or D_ij_ = 1, P_ji_ = proportion of samples in which X_j_ dominated X_i_ or D_ij_ = 0, P_noij_ = proportion of samples in which dominance between X_i_ and X_j_ could not be established or D_ij_ = 0.5, Reproducibility = or proportion of bootstrap samples that agree with the sample results. For each pair, only one order is shown in the table, such that P_ij_ > P_ji_.*

With regard to complete dominance, the results confirmed those obtained by previous analyses. The means of the dominance values were close to one, and the reproducibility was good. Only in the case of dominance of habits (*X*_5_) on attitude (*X*_3_), the dominance values (0.7830) and reproducibility (59.7%) were lower. Even in the case of conditional dominance, the results confirmed those previously obtained, although the means of dominance values and reproducibility were lower. Finally, general dominance confirmed previous results and reduced the number of indeterminacies except for the dominance of attitude (*X*_3_) on financial incentives (*X*_6_), which show low values of dominance (0.5100) and reproducibility (51%), indicating that in half of all samples, dominance between these two variables is undetermined.

## Discussion

This study aimed to establish the relative weight of habits, financial incentives, attitudes, norms, and PBC in influencing the intention of using a bicycle for daily commuting using the dominance analysis approach. The literature on this topic usually uses the traditional regression model approach (e.g., Caballero et al., 2019) instead of dominance analysis. However, dominance analysis is better than other approaches in determining the relative importance of predictors. Usually, researchers intend to explain an outcome variable from various predictors. This process can be divided into two phases: model selection and predictor comparison (Budescu, 1993; Azen et al., 2001). In the first phase, researchers identify the model and consider various predictors that better describe the outcome variables. The second phase evaluates predictor importance by comparing the contributions made by each independent variable included in the model in predicting the dependent variable. Compared to multiple regression, the dominance analysis approach can complete the second stage by clearly defining the importance of predictors. While other approaches, such as regression, infer the importance of predictors by interpreting a statistical coefficient, dominance analysis has a clear definition of importance that facilitates the identification of predictor importance: an independent variable is considered more important than another if it contributes more than the other to the prediction of the dependent variable (Azen and Budescu, 2003). Moreover, by assigning a weight to each variable, this procedure could be closer to how people decide whether or not to use the bike.

The results of our study indicated that habits are the stronger variable that influences the intention to use a bicycle for daily commuting, followed by attitude and incentives, which seem to play almost the same role. Finally, the less important variables related to intention are both types of norms and control. Concerning the role of habit, the results are similar to those of other studies carried out in different contexts (Rondinella et al., 2012; Muñoz Lopez et al., 2013). While, considering financial incentives, this variable has shown good power in predicting intentions, which is contrary to previous studies (Savelkoul and Peutz, 2017) and similar to other ones (Bamberg and Schmidt, 1998; Hunecke et al., 2001; Thøgersen, 2009; Martin et al., 2012; de Krujif et al., 2018; Moser et al., 2018; Ciccone et al., 2021). Financial incentives are nearly equivalent to the attitude toward bicycle use, thus confirming its important role as a predictor of bicycle use (Dill and Voros, 2007; Heinen et al., 2011; Milkovic and Stambuk, 2015; Frater et al., 2017; Yu et al., 2018). It is interesting to observe the relationship between financial incentives and subjective norms: in our study, in opposition to other researches (Hunecke et al., 2001; Riggs, 2017), financial incentives dominate subjective norms. It is an important result if we consider the Italian context: this means that the kind of incentive used by the government is quite efficient to determine a change in the intention, despite the absence of strong support from significant others. Moreover, it should be useful to reflect on the relation between habits and incentives: other studies have observed their interplay in particular moments as relocation, showing the strength of incentives in changing behavior (Bamberg, 2006). However, in our research, habits dominate incentives, stressing the importance of practice creation before financial incentives.

In relation to PBC, unlike other studies (Caballero et al., 2014; de Souza et al., 2014; Acheampong and Siiba, 2018; Si et al., 2020), the present research shows a very low impact of this variable in determining intention, together with subjective norms. These results may be because Italy is less “bike-friendly” than other countries. For example, in the 2019 Bicycle Cities Index (Coya, 2019) results, which analyzed the conditions for cycling in 90 cities across the world, Italian cities are ranked toward the bottom of the ranking (Milan is in 65th place and Rome is in 70th place). However, these results may also depend on the sample’s composition, which was not representative of the Italian population and was mainly composed of female participants. Obviously, the sample was affected by the self-selection, probably because the data were collected by an online questionnaire distributed over social networks, and the participation was voluntary. It would be desirable to repeat the study with a well-balanced sample and identify subgroups of populations to explore possible differences between them (Winters et al., 2017). Furthermore, regarding PBC, we do not have information about the conformation of the territory in which the participants live. Indeed, using a bike could be simpler living on plains than living on mountains or hills (see, for example, Heinen et al., 2010; Tyndall, 2020). Further research should also consider this aspect of behavioral control. Finally, the last limitation is the self-report measure used, which involves the possibility of response bias.

However, despite these limitations, this study suggests that bicycle use is affected by various factors that need integration, as suggested in other contexts (e.g., Savan et al., 2017) and provides some important indications for promoting bicycle use by acting on variables that showed the greatest predictive power. Both habits and attitudes underly the importance of establishing a bike use culture in cities. Thus, both habits and attitudes should be supported through communication campaigns focusing on positive aspects of using cycles for daily commuting (e.g., time savings and/or health benefits). Regarding financial incentives, they seem to be an interesting method of improving the use of bicycles as a means of transportation. It would be advisable to offer financial incentives regularly and not as an exception; for example, the incentives given by the Italian government led to a 17% increase in bicycle sales in 2020 compared to the previous year (ANCMA, 2020). The role of incentives leads us to an important consideration: financial incentives together with attitude could represent an important method of promoting the shift toward bicycles as a means of transportation. The Italian government has reserved many resources to increase bicycle purchases to create and maintain healthy mobility habits.

In this study, the dominance analysis was used to establish the relative importance of the predictors; however, similar results could be obtained using the relative weight analysis (Johnson, 2000). To calculate the relative importance of the predictors, dominance analysis performs a series of regressions; on the other hand, the relative weight analysis transforms the independent variables into a new set of independent variables that are orthogonal to each other but still correlated with the original ones. Despite the differences in statistical rationale, both analyses arrive at nearly identical results (Johnson, 2000; LeBreton et al., 2004).

Future research could also provide insights on behavior maintenance in individuals who used financial incentives to buy a bicycle. Furthermore, given the limited effects of the norms and perceived control, probably due to the Italian context and the sample, the study should be repeated considering other samples and more “bike-friendly” contexts.

## Data Availability Statement

The raw data supporting the conclusions of this article will be made available by the authors, without undue reservation.

## Ethics Statement

The studies involving human participants were reviewed and approved by Internal Ethic Review Board (IERB), Department of Educational Sciences (Psychology Section), University of Catania. The patients/participants providedtheir written informed consent to participate in this study.

## Author Contributions

VB: conceptualization, methodology, data collection, and writing—original draft. ZH: supervision, methodology, data analysis, and writing—original draft. FS and CD: writing—reviewing and editing. All authors contributed to the article and approved the submitted version.

## Conflict of Interest

The authors declare that the research was conducted in the absence of any commercial or financial relationships that could be construed as a potential conflict of interest.

## Publisher’s Note

All claims expressed in this article are solely those of the authors and do not necessarily represent those of their affiliated organizations, or those of the publisher, the editors and the reviewers. Any product that may be evaluated in this article, or claim that may be made by its manufacturer, is not guaranteed or endorsed by the publisher.

## Footnotes

1 PBC has been differently conceptualized in the various studies considered, and it refers both to the perception of infrastructure (cycle paths, cycle routes, bike parking spaces) and personal self-efficacy.
