# Supplementary material for: Comparing the Relative Importance of Predictors of Intention to Use Bicycles
Source: Front Psychol. 2022 Feb 17;13:840132. doi: 10.3389/fpsyg.2022.840132 (PMC8891601; doi:10.3389/fpsyg.2022.840132)
Supplement: Supplementary file 1 [file Table_1.DOCX]

QUESTIONNAIRE

[*The names of the measures are indicated in brackets*]

We invite you to participate in research on the use of bicycles, coordinated by prof. Zira Hichy (Department of Educational Sciences, University of Catania). This research aims to understand factors influencing the use of bicycles for daily commuting. To participate, you only have to complete a 10 minutes questionnaire. Participation is voluntary and free, and you can withdraw from the study at any time. The processing of the data collected will be conducted in such a way as to eliminate any reference that may allow the connection of individual statements to a specific person. The research results will be published in summary form, and in no case will they be attributable to individuals.

For any information and clarification on this study or any need, please contact Zira Hichy (z.hichy@unict.it).

We ask you to answer the following statements. We invite you to circle the number corresponding to your opinion for each of them.

- How many of your friends/relatives use a bike to travel around the city? [Descriptive norms]

| 1 | 2 | 3 | 4 | 5 | 6 | 7 |
| --- | --- | --- | --- | --- | --- | --- |
| None | Few | Some | Half | Many | Almost all | All |

- People important to me think I should ride a bike more often. [*Prescriptive norms*]

| 1 | 2 | 3 | 4 | 5 | 6 | 7 |
| --- | --- | --- | --- | --- | --- | --- |
| Strongly disagree | Disagree | Somewhat disagree | Neither agree nor disagree | Somewhat agree | Agree | Strongly agree |

- Using a bike for daily commuting is for me (for each pair of adjectives, we ask you to circle the number corresponding to your opinion). [*Attitude toward behavior*]

| Difficult | 1 | 2 | 3 | 4 | 5 | 6 | 7 | Easy |
| --- | --- | --- | --- | --- | --- | --- | --- | --- |
| Challenging | 1 | 2 | 3 | 4 | 5 | 6 | 7 | Simple |
| Expensive | 1 | 2 | 3 | 4 | 5 | 6 | 7 | Cheap |
| Unsafe | 1 | 2 | 3 | 4 | 5 | 6 | 7 | Safe |
| Dangerous | 1 | 2 | 3 | 4 | 5 | 6 | 7 | Comfortable |
| Boring | 1 | 2 | 3 | 4 | 5 | 6 | 7 | Exciting |
| Strenuous | 1 | 2 | 3 | 4 | 5 | 6 | 7 | Relaxing |
| Slow | 1 | 2 | 3 | 4 | 5 | 6 | 7 | Fast |
| Useless | 1 | 2 | 3 | 4 | 5 | 6 | 7 | Useful |

- I see many difficulties in riding a bike. [*Perceived behavioral control, reverse coded*]

| 1 | 2 | 3 | 4 | 5 | 6 | 7 |
| --- | --- | --- | --- | --- | --- | --- |
| Strongly disagree | Disagree | Somewhat disagree | Neither agree nor disagree | Somewhat agree | Agree | Strongly agree |

- The infrastructure of my city is adequate for getting around by bicycle. [*Perceived behavioral control*]

| 1 | 2 | 3 | 4 | 5 | 6 | 7 |
| --- | --- | --- | --- | --- | --- | --- |
| Strongly disagree | Disagree | Somewhat disagree | Neither agree nor disagree | Somewhat agree | Agree | Strongly agree |

- Before the lockdown, how often did you use your bicycle for daily commuting? [*Habits*]

| 1 | 2 | 3 | 4 | 5 | 6 | 7 |
| --- | --- | --- | --- | --- | --- | --- |
| Never | Rarely | Sometimes | Half the time | Often | Almost always | Always |

- Government incentives for purchasing a bike encourage me to buy it. [*Financial incentives*]

| 1 | 2 | 3 | 4 | 5 | 6 | 7 |
| --- | --- | --- | --- | --- | --- | --- |
| Strongly disagree | Disagree | Somewhat disagree | Neither agree nor disagree | Somewhat agree | Agree | Strongly agree |

- I think I will use the government incentives for purchasing bicycles. [*Financial incentives*]

| 1 | 2 | 3 | 4 | 5 | 6 | 7 |
| --- | --- | --- | --- | --- | --- | --- |
| Strongly disagree | Disagree | Somewhat disagree | Neither agree nor disagree | Somewhat agree | Agree | Strongly agree |

- I intend to start using a bike for daily commuting. [*Intention*]

| 1 | 2 | 3 | 4 | 5 | 6 | 7 |
| --- | --- | --- | --- | --- | --- | --- |
| Strongly disagree | Disagree | Somewhat disagree | Neither agree nor disagree | Somewhat agree | Agree | Strongly agree |

We ask you for some personal information:

- Gender: Male ⬜ Female ⬜
- Age: __________
- Country of birth: ________________________________
- Region of residence: ________________________________
