# Supplementary material for: Comparing the Relative Importance of Predictors of Intention to Use Bicycles
Source: Front Psychol. 2022 Feb 17;13:840132. doi: 10.3389/fpsyg.2022.840132 (PMC8891601; doi:10.3389/fpsyg.2022.840132)
Supplement: Supplementary file 2 [file Table_2.DOCX]

Residence of the participants (N = 294)

| Macro-Area | Region | N |
| --- | --- | --- |
| Central Italy  (n = 62) | Abruzzo | 1 |
|  | Latium | 45 |
|  | Marches | 1 |
|  | Tuscany | 12 |
|  | Umbria | 3 |
| Northern Italy  (n = 165) | Emilia Romagna | 32 |
|  | Friuli-Venezia Giulia | 10 |
|  | Liguria | 13 |
|  | Lombardy | 58 |
|  | Piedmont | 26 |
|  | Trentino-Alto Adige | 2 |
|  | Veneto | 24 |
| Southern Italy and Island  (n = 67) | Basilicata | 1 |
|  | Calabria | 2 |
|  | Campania | 10 |
|  | Molise | 2 |
|  | Apulia | 5 |
|  | Sardinia | 4 |
|  | Sicily | 43 |
